# Identification of natural selection in genomic data with deep convolutional neural network

**DOI:** 10.1186/s13040-021-00280-9

**Published:** 2021-12-04

**Authors:** Arnaud Nguembang Fadja, Fabrizio Riguzzi, Giorgio Bertorelle, Emiliano Trucchi

**Affiliations:** 1 Dipartimento di Matematica e Informatica, University of Ferrara, Via Saragat 1, Ferrara, I-44122 Italy; 2 Dipartimento di Scienze della Vita e Biotecnologie, University of Ferrara, Via Luigi Borsari 46, Ferrara, I-44121 Italy; 3 Dipartimento di Scienze della Vita e dell’Ambiente, Marche Polytechnic University, Via Brecce Bianche, Ancona, I-60131 Italy

**Keywords:** Genomic data, Inference of natural selection, Deep Learning, Convolutional Neural Networks

## Abstract

**Background:**

With the increase in the size of genomic datasets describing variability in populations, extracting relevant information becomes increasingly useful as well as complex. Recently, computational methodologies such as Supervised Machine Learning and specifically Convolutional Neural Networks have been proposed to make inferences on demographic and adaptive processes using genomic data. Even though it was already shown to be powerful and efficient in different fields of investigation, Supervised Machine Learning has still to be explored as to unfold its enormous potential in evolutionary genomics.

**Results:**

The paper proposes a method based on Supervised Machine Learning for classifying genomic data, represented as windows of genomic sequences from a sample of individuals belonging to the same population. A Convolutional Neural Network is used to test whether a genomic window shows the signature of natural selection. Training performed on simulated data show that the proposed model can accurately predict neutral and selection processes on portions of genomes taken from real populations with almost 90% accuracy.

## Introduction

The technological advancements in DNA/RNA sequencing is sustaining an unprecedented growth in the amount of genomic data. At the same time, population geneticists are increasingly interested in finding new and more accurate models to understand the evolutionary processes generating the observed patterns [1] and predict future changes [2]. In particular, many studies addressed the problem of quantifying the relative contributions of natural selection and random drift in shaping the genetic variation observed in living organisms [3].

Recently, many authors have shown that inferences regarding patterns and distributions of the effects of selection in genes and genomes can offer the key to understanding the functions of different genomic regions. For example, in the human genome, Mendelian-disease genes are largely under purifying selection whereas genes responsible for complex traits could be subjected to either purifying or positive selection [4]. Therefore, it might be possible to identify alleged genetic factors related to diseases or to relevant traits by identifying regions in the human genome that are currently under selection [3]. Moreover, in wild or domesticated species, uncovering the pattern of natural selection in their genomes can help identify the genomic basis of their adaptation, e.g. [5]. Inferring the signature left by natural selection is typically addressed comparing the theoretical expectations predicted in case of random drift (neutral evolution) or natural selection with the real data, and the comparison is based on summary statistics of genetic variability [6, 7].

New advancements in Big Data Analysis are essential to meet the computational challenge that genomics poses [8]. Modern Machine Learning methods are able to exploit very large datasets, often represented by images, to find hidden patterns and make accurate predictions. The interaction between biological knowledge and Machine Learning architectures is very promising for searching hidden patterns in large amounts of genomic data [9]. Instead of using summary statistics or mathematical models of population genetics, we rely on Machine Learning techniques to identify patterns of genetic variability [10, 11]. Interestingly, genetic data can easily be converted into images and can be analyzed by Convolutional Neural Networks, an innovative Machine Learning technology for image classification [12]. Experiments performed on simulated genetic data show that Convolutional Neural Networks can accurately predict Neutral and Natural selection phenomena on real genetic data.

The paper is organized as follows: after a brief presentation on Convolutional Neural Networks and genetic models in the Convolutional neural networks and Genetic models and machine learning sections respectively, Related work section presents related work. In the Dataset generation section describes how the genetic data was generated and experiment on simulated data is presented in the Experiments section. An application to real genomic data is presented in the Application to real data and Conclusion sections concludes the paper.

## Convolutional neural networks

Machine Learning (ML) [13] and Deep Learning (DL) [14] are fields of Artificial Intelligence which study algorithms for analysing and inducing knowledge from data. They are defined as a set of methods that can automatically identify patterns in data and then use these patterns to predict future (unseen) data or to perform decision-making under uncertainty [15]. Recently, a DL algorithm called Convolutional Neural Network (CNN) [16] has shown a good ability to extract patterns from data, such as images. CNNs are considered the state of the art for solving classification problems and have been successfully applied to image classification and object detection [17].

**Fig. 1:**
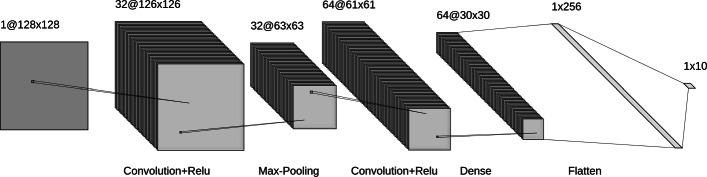
Convolutional Neural Network

**Fig. 2:**
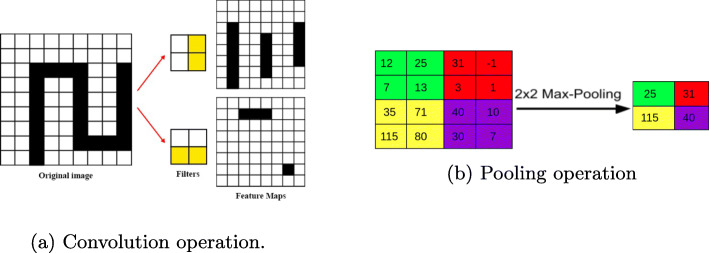
Convolution and pooling operations

**Fig. 3:**
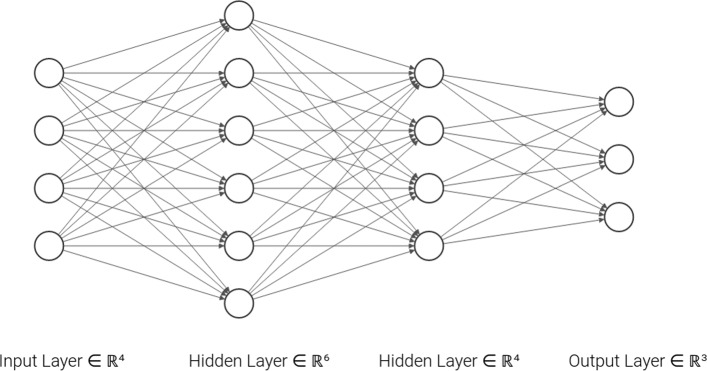
Fully connected neural network

A CNN, see Fig. 1, is divided into two parts: the first part, *feature learning*, extracts patterns or features from the image while the second part, *classification*, classifies the image based on the extracted features. The first part relies on two operations: the *convolutional* operation, Fig. 2a scans the image from the top to the bottom to extract possible features using a certain number of filters also called kernels. Each filter extracts a different type of feature. After convolution, *pooling* operation, Fig. 2b, is applied to downsample the extracted features and reduce the complexity of the representation. It also scans the features and performs an operation (generally Maximum) which summarizes a set of values into one. A function, called *activation function*, e.g the Rectified Linear Unit (ReLu), is generally applied to the output of each convolution operation to introduce nonlinearity in the model. The convolution and pooling operations are organized into layers. The first layers identify common features like corners and edges. The deeper the layer the more complex the features extracted are. The *classification* portion of the network, [18], is a shallow (dense) artificial neural network also called *fully connected neural network*, see Fig. 3, in which artificial neurons are organized into layers. Each neuron receives inputs from all neurons in the previous layer, and performs a linear combination on these inputs whose result is passed through an activation function, generally ReLu. The first layer is the *input layer* which represents features extracted in the convolutional part and the last layer is the *output layer* yielding the predicted responses. The number of neurons in the output layer is equal to the number of classes to identify or to the number of objects to detect. Intervening layers of the classification portion are called *hidden layers*.

Once the architecture of the network is defined, it should be trained to perform a certain task. In the case of binary classification (two classes), training a CNN means repeatedly adjusting, for example by means of gradient descent [19] and back-propagation [20], both the filters in the convolutional layers and the weights in the dense layers using a set of examples called *training set*. These adjustments are done such that eventually the model is able to correctly classify almost all the examples in the training set and hopefully acquires the ability to correctly classify new examples, called *generalization capacity*.

Training a neural network is also repeatedly updating its weights in order to minimize an objective or loss function. The loss function measures how well a network behaves at each iteration. It recapitulates in a single value the differences between the expected classes of examples in the training set and the ones provided by the network. The training accuracy of the network is the fraction of examples correctly classified with respect to the whole examples. Training a network is therefore gradually updating its weights which progressively reduces the loss (or increases the accuracy) in order to correctly classify almost all examples in the training.

The standard gradient descent algorithm computes gradients at each iteration using all examples in the training set. If the training set is large, the algorithm can converge very slowly. To avoid slow convergence, gradients can be computed using a single example, randomly selected in the training set. Even if in this case the algorithm can converge quickly, it is generally hard to reach high training set accuracy. A compromise often used is the mini batch stochastic gradient descent (SGD) [21]: at each iteration a mini batch of examples from the training set is randomly sampled to compute the gradient. The training process is divided into *epochs*. An *epoch* occurs when all the examples in the training are used. This method usually provides fast converge and high accuracy.

Since the construction and the training of a network rely on a set of parameters named hyper-parameters, a *validation set* of examples is often useful to find the hyper-parameters values which lead to better generalization. If the model provides acceptable accuracy (or loss) on the training set and bad on the validation set, this phenomenon is called overfitting. Many techniques are available to avoid overfitting. A technique called Dropout [22] drops a fraction of neurons in some layers at training time. This prevents a net with lot of weights to overfit on the training data. Other techniques to avoid overfitting called *L*_1_ and *L*_2_ regularization [23] add another term (that depends on the weight) to the loss function in order to favor small weights (∼0). Small weights can then be dropped during the evaluation of the network. Then the accuracy of the model is assessed by running the model on a set of unseen examples called *test set*.

## Genetic models and machine learning

The classic model of selection in population genetics includes two alleles, typically denoted by *A* and *a*, which are alternative variants of a DNA fragment present in a specific position of the genome. *A* and *a* can refer to a DNA fragment composed by a single or by multiple nucleotides. *Natural selection* occurs when the fitness (i.e. the probability of survival and reproduction, indicated with *w*) of the three genotypes that can occur in a diploid individual (*AA*, *Aa* and *aa*; diploid organisms have always two copies of each chromosome) are different. There are different types of selection which can be simplified as *positive* (directional), *balanced*, and *negative* (purifying) selection. Positive selection occurs if the fitness of a given genotype is higher than the others, i.e. if *w*_*AA*_>*w*_*Aa*_>*w*_*aa*_ or *w*_*AA*_<*w*_*Aa*_<*w*_*aa*_, and tends to get fixed in the population while the other allele is lost. Balanced selection occurs when selection favors the maintenance of diversity within a population ((*w*_*AA*_<*w*_*Aa*_>*w*_*aa*_). Negative selection is the selection against one of the two alleles which appears as disadvantageous. A mutation that does not affect the fitness of the individual in which it occurs (and therefore *w*_*AA*_=*w*_*Aa*_=*w*_*aa*_), is called *neutral*; more generally, neutrality describes the condition in which the locus under consideration is not affected by selection [3]. As genetic loci are physically arranged along a chromosome, the effect of selection can extend on the two sides of the selected locus and interfere with the selective (or neutral) trajectory of allele frequencies at nearby loci. Such interference (i.e., due to linkage disequilibrium, that is the non-random association of alleles at different loci) can be released by recombination whose probability scales proportionally with the physical distance between loci. The extent of the signature of selection in a genomic region harbouring a positively selected allele depends on its differential fitness (selective coefficient) throughout its trajectory, from appearance to fixation, on the recombination rate in that region, and on the population size. Given that drift can also lead to fixation or loss of alleles due to random fluctuations of the allele frequencies, and that genetic drift overwhelms selection in small populations, identifying the signature of selection from that left by drift is one of the most challenging tasks in population genetics.

The purpose of this work is to predict whether a genomic region shows a pattern which is more consistent with a natural selection or a neutral model, assuming that all the other parameters of the evolutionary process are constant. Genomic regions simulated with both models are represented as black and white images by converting the 0,1 matrices of genomic data as described below (Dataset generation section). The rows of these matrices represent the mutations of nucleotide bases of a certain genomic sequence for each individual and the columns represent individuals belonging to a population. In this way it is possible to summarize in a single image information on the allelic frequencies of mutations related to a specific genomic sequence in a population. CNNs are then used to analyze images representing sections of the genome and classify them as produced by a neutral or a natural selection model.

## Related work

In [12], the authors propose a program called ImageGene which identifies positive selection on genetic data using CNNs. Both ImaGene and our system rely on the *ms* program [24] to generate the neutral genetic data. However, to generate selection data we use a modified version of the *ms* program called *mssel* made available by [24] as explained in the Dataset generation section, while ImaGene uses the *msms* program [25]. Both *mssel* and *msms* are modified versions of the *ms* program. Similar to our approach, ImaGene converts genetic data into images and uses a CNN to extract and classify patterns from them. Our approach strongly differs from ImaGene in the way the genetic data are generated and in the way they are converted into images. For example, while we generated binary matrices of the same size including a fixed number of sites regardless of whether they are variant or invariant, the ones generated by ImaGene are of different sizes as they include only variable sites. ImaGene then transforms all binary matrices into a 128x128 pixel image with considerable loss of information. Unlike [12], we converted a single binary matrix into a binary image with the same size which maintains all the relevant patterns useful for its classification. Indeed, we obtain high performance in terms of accuracy using less examples than those used in ImaGene.

In [26] the authors use a supervised Machine Learning approach, called support vector machine (SVM), to discriminate between genomic regions experiencing purifying selection and those free from a selective constraint using population genomic data. Different from our approach, the authors of [26] take as input the Site Frequency Spectrum (SFS)^1^ of individuals from the Phase I release of 1000 Genomes Dataset which consist of 14 population samples from diverse global locations. In [27] the authors used an unsupervised ML algorithm called hidden Markov models (HMMs) to detect regions of genomes under positive or negative selection. For an overview on how to apply Machine Learning to genetic data see [28].

## Dataset generation

**Fig. 4:**
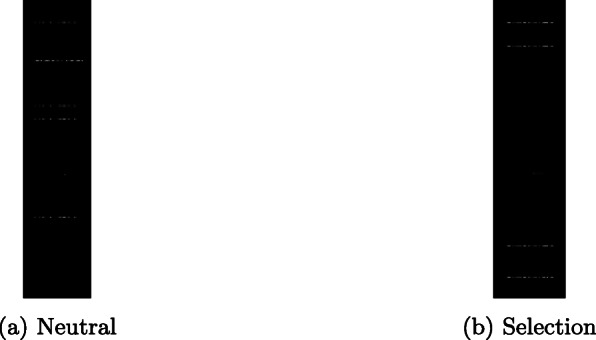
Neutral and Selection

A dataset was created using the *ms* program [24] which allows the simulation of genetic data based on a demographic model defined by the user without selection so that the trajectory of the allele frequencies is only driven by genetic drift (i.e., Neutral). Its modified version *mssel* (made available by [24]) allows the effect of natural selection to be added to the demographic model (Selection). Using this program, we simulated DNA sequences of defined length for a sample of individuals taken from a population with a fixed size. In each simulation, the sample of DNA sequences can be linked together by a coalescence tree that goes back to a common ancestor in previous generations [29]. Along this tree, random mutations may appear that modify a base of the DNA sequence with a probability set by the user. The final variability pattern of this simulated sample of DNA sequences depends on the population size, the probability of mutation and the demographic history set in the model. In the case of the *mssel* program, the pattern of variability of the DNA sequences in the sample will also depend on the intensity of selection, another parameter of the model. In both cases, the program produces arrays of DNA sequences, where one dimension corresponds to the length of the sequence while the other to the number of simulated individuals. Since it is a simulation, it is easy to keep track of the ancestral state and the mutated one for each single base of the DNA sequence and then report the sequences as strings of "0" (ancestral state) and "1" (derived state, i.e. a mutation occurred in the base). The matrix representing the individual sequences can then be stored as a binary matrix (composed of 0 and 1) which can be easily converted into a binary image, see Fig. 4. The output of each simulation (either from ms or mssel) is parsed as a random alignment of sampled copies (i.e., 48) of the simulated genetic region, with length as the number of base pairs in the simulation settings (e.g., 1,000 bp). Importantly, we include both variant and invariant sites in the alignment to produce genetic regions of fixed length. We then transpose the alignment into a matrix of 1000 rows and 48 columns and convert it into a black and white png file using a lossless conversion (see Fig. 4). Matrices are in fact converted end to end to images by mapping each 0 value to a black pixel and each 1 value to a white pixel. Therefore, the resulting images are of size 1000x48 and maintain all the information from the simulated genetic regions.

In particular in our case study, using the script available in the experiment repository dataset_creator.py^2^ the data for both Selection or Neutral models were generated by iteratively running the following command ms2raster.py -bp lines -s number-matrices -l mode -selstr intensity-of-selection -p path-dataset -i number-individuals, which generates binary matrices as defined previously for training, validation and testing. The parameter *bp* indicates the length in bases of the DNA sequences to be simulated and corresponds to the number of rows of the generated matrices to be generated. We set *bp*=1000. The parmeter *-s* provides the total number of matrices. The parameter *-l* indicates the mode, *selection*, *neural*. The *selstr* (selection strength) parameter controls the intensity of the selection in favor of a certain allele. Our simulations were performed considering an effective population size of 80,000 individuals and *selstr*=0.005. In addition, we used a rather large effective population size which is expected to downplay the effect of genetic drift as compared to selection. The parameter *-p* indicates path to the *ms* and *mssel* binaries. Finally the parameter *-i* represents the number of individuals, which we set to 24. The generated matrices are therefore of dimension [1000×48], where the lines represent consecutive nucleotides in the genomic sequence and the columns represent the chromosomes of all sampled individuals. Note that the number of columns is twice the number of individuals since each diploid individual has 2 chromosomes (and therefore two copies of each nucleotide in the sequence). The mutation and the recombination rates were both set to 10*e*−8 and *bp*^−1^.

**Fig. 5:**
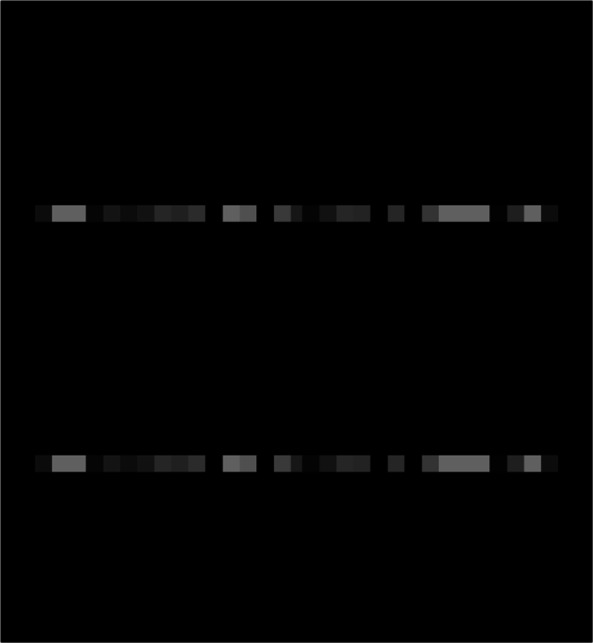
Portion of Fig. 4b

**Fig. 6:**
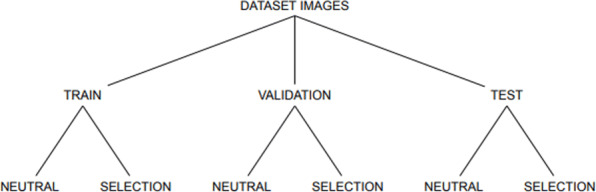
Dataset sub-division

Since CNNs operate on images, the generated matrices were converted into binary images. Examples of images representing neutral and selection phenomena are shown in Fig. 4a and b respectively. Figure 5 shows a portion of Fig. 4b. The script dataset_creator.py creates a user-defined number of dataset (already converted into images) for each model (Neutral, Selection) and for each of the three sets: TRAIN, VALIDATION, TEST as depicted in Fig. 6.

## Experiments

Experiments^3^ were performed using Python as programming language and Keras (with TensorFlow as the backend) as Framework. Experiments were performed using the K80 NVIDIA GPU.

Initially, we considered a network consisting of 2 convolutional layers, each with a pooling layer, and two densely connected layers, of which the first has a fairly high number of units (1024) and the last has a single unit (as we are dealing with a binary classification problem). ReLu was chosen as the activation function for every layer except for the last layer in which the Sigmoid activation function was used.

**Fig. 7:**
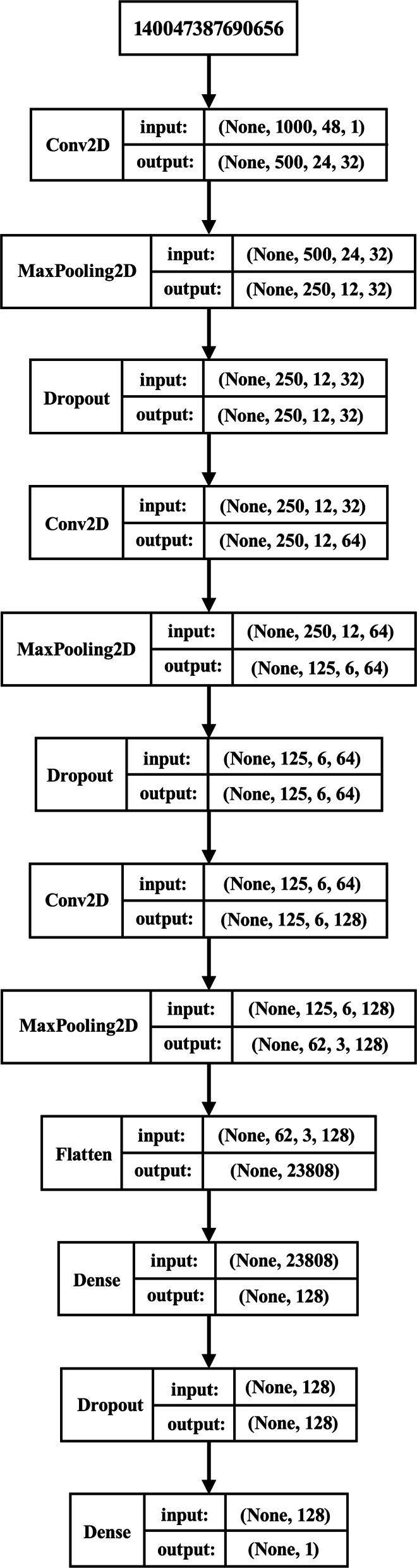
Model

Several experiments showed that both on small and large datasets the model presented clear signs of overfitting probably due to a lot of weights in model and shallow patterns extracted from the images. This led to performing different experiments on different datasets. The datasets are recapped in Table 1 where 10k means 10,000 and ds means dataset. Note that an equal number of images were generated for both Selection and Neutral in all datasets. To avoid overfitting while extracting relevants patterns on the images, the structure of the model was reviewed as follows: we expanded the model, as shown in Fig. 7. Further convolutional layers were added (with 32, 64 and 128 filters of dimension [10, 10] and stride (2, 2) respectively) to enable relevant patterns extraction. To prevent overfitting, several dropout layers (with rate 0.4) were also added. The units of the first densely connected layer where also reduced, from 1024 to 128. Initially, Adam was used as the optimizer but led to unsatisfactory results. Then, SGD with momentum = 0.9 was used as optimizer [30].

**Table 1:** Datasets

Dataset	Train	Validation	Test
ds1	100k	20k	20k
ds2	50k	30k	30k
ds3	50k	10k	10k
ds4	10k	1k	1k

Regarding the hyperparameters, a mini-batch of 100 images were used in the case of the ds1 dataset, while 50 images were used for the remaining datasets. This led to a high accuracy from the initial training epochs on both Training and Validation sets. Different learning rates were also tested for each dataset to try to obtain high accuracy on the Validation set with a reduced number of epochs. Tables 2, 3, 4 and 5 report the results of the experiments using 10 epochs, the accuracies on the test set are highlighted in bold.

**Table 2:** Results with 10 epochs on ds1 dataset

L. rate	Train acc.	Val. acc.	Test acc.	Train Loss	Val. Loss	Test loss	Time(s)
1.0e-3	0.956	0.954	0.954	0.117	0.138	0.138	1396
5.0e-4	0.950	0.955	0.955	0.133	0.141	0.141	1392
1.5e-4	0.940	0.944	0.944	0.167	0.172	0.172	1395
1.0e-5	0.938	0.943	0.943	0.175	0.177	0.177	1417

**Table 3:** Results with 10 epochs on ds2 dataset

L. rate	Train acc.	Val. acc.	Test acc.	Train Loss	Val. Loss	Test loss	Time(s)
1.0e-3	0.964	0.972	0.972	0.097	0.084	0.084	641
5.0e-4	0.959	0.958	0.958	0.112	0.116	0.116	642
1.5e-4	0.950	0.960	0.960	0.136	0.122	0.122	635
1.0e-5	0.947	0.957	0.957	0.150	0.130	0.132	647

**Table 4:** Results with 10 epochs on ds3 dataset

L. rate	Train acc.	Val. acc.	Test acc.	Train Loss	Val. Loss	Test loss	Time(s)
1.0e-3	0.962	0.977	0.977	0.099	0.075	0.075	581
5.0e-4	0.958	0.960	0.960	0.110	0.108	0.108	587
1.5e-4	0.951	0.957	0.957	0.136	0.122	0.122	589
1.0e-5	0.946	0.955	0.955	0.150	0.139	0.139	581

**Table 5:** Results with 10 epochs on ds4 dataset

L. rate	Train acc.	Val. acc.	Test acc.	Train Loss	Val. Loss	Test loss	Time(s)
1.0e-3	0.890	0.944	0.940	0.265	0.192	0.189	118
5.0e-4	0.870	0.941	0.920	0.300	0.211	0.241	115
1.5e-4	0.790	0.900	0.870	0.450	0.370	0.398	117
1.0e-5	0.800	0.880	0.869	0.430	0.377	0.383	116

From these tables we can see that using datasets of different sizes and different learning rates led to very similar training accuracy. Dataset ds4 (i.e. 5*k* for Neutral class and 5*k* for Selection class) is the one that produces the lowest accuracy, but still in line with the other results, since images in the datasets are still very similar. It can also be observed that dataset ds3 provides a good balance between number of samples, accuracy and training time. Note that, the value of the learning rate that leads to higher accuracy is 0.001.

**Fig. 8:**
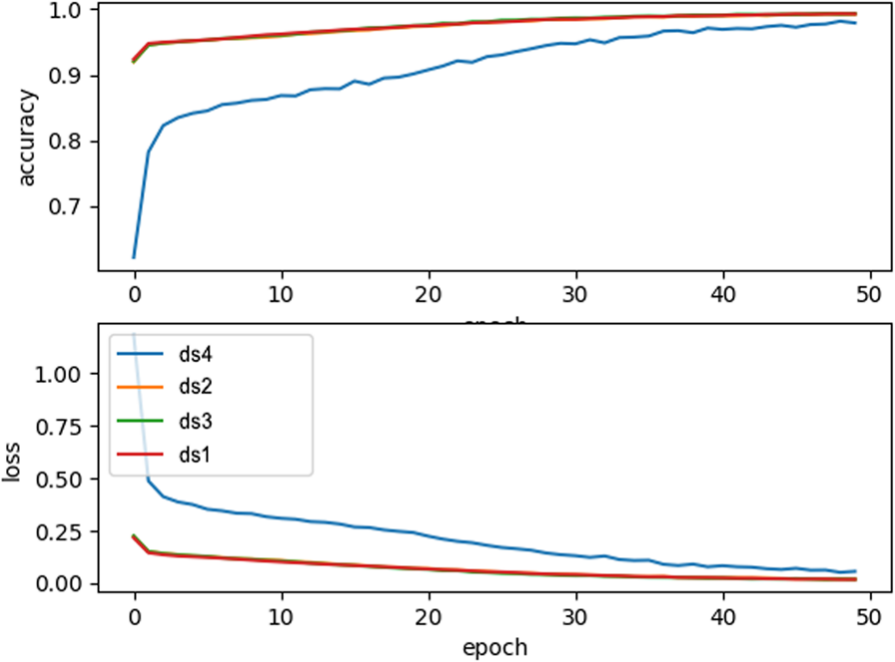
Training Accuracy and loss of datasets over 50 epochs

In order to increase the accuracy, we first increased the number of training epochs. Further experiments with 50 epochs were performed and led to a higher accuracy. As depicted in Fig. 8, the training accuracy and the training loss of the model trained on the various datasets during 50 epochs are very similar. Observe that dataset ds4 initially deviates from the others but converges similarly when the maximum number of epochs is reached.

**Fig. 9:**
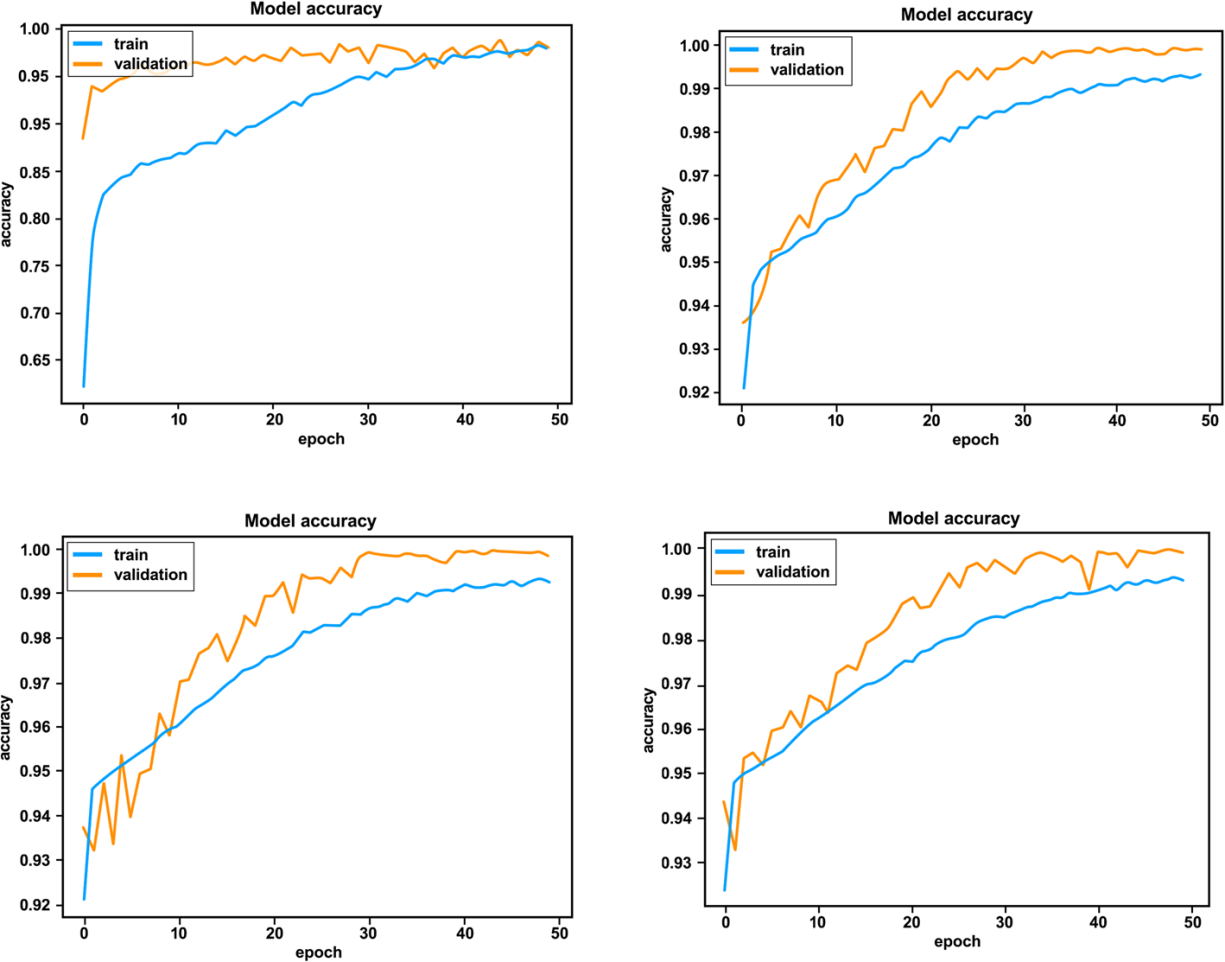
Accuracies with 50 epochs on the datasets ds1(top left), ds2 (top right), ds3 (bottom left) and ds4(bottom right)

**Fig. 10:**
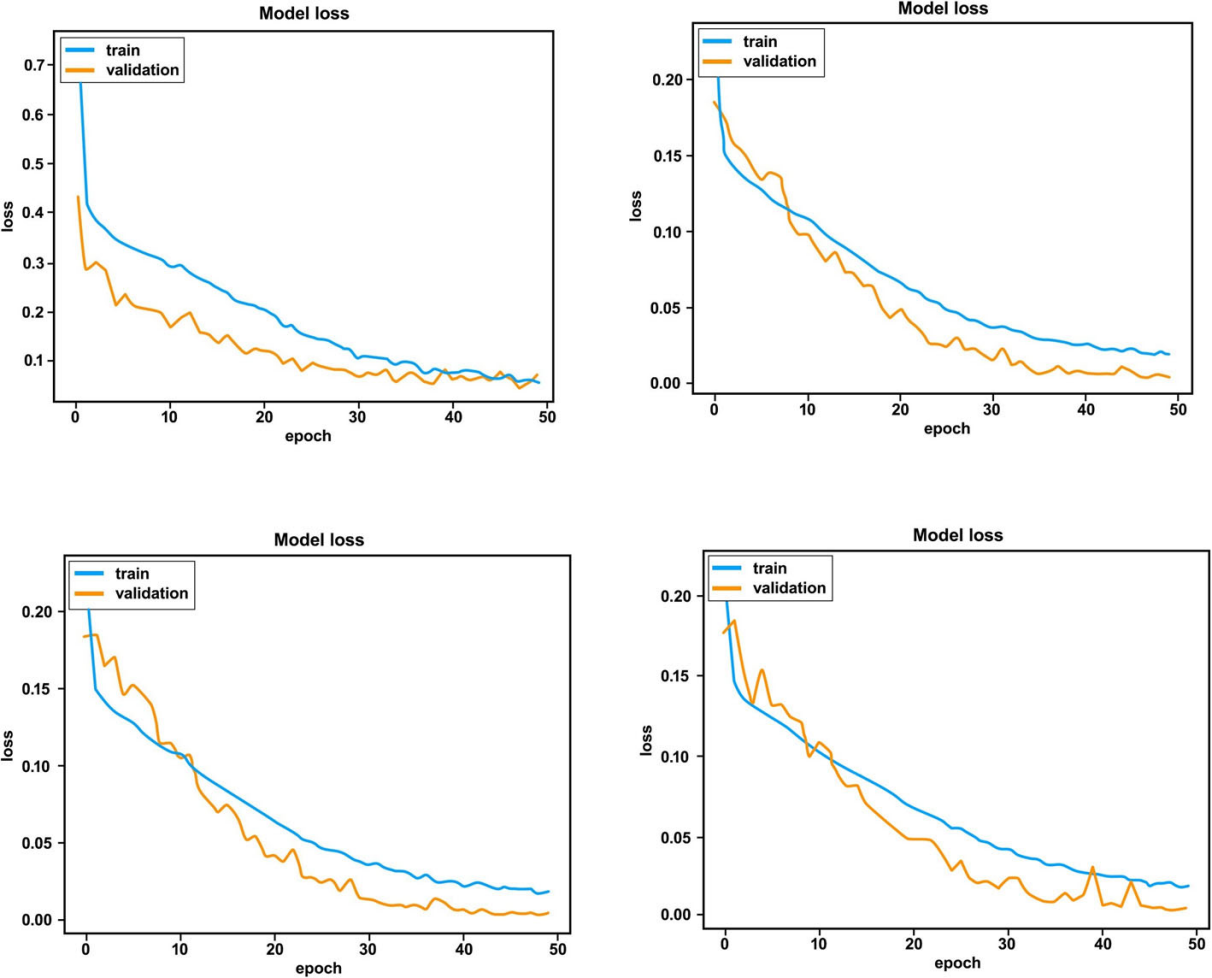
Losses with 50 epochs on the datasets ds1(top left), ds2 (top right), ds3 (bottom left), ds4(bottom right)

Figures 9 and 10 show the accuracy and loss of the training and validation set over 50 epochs respectively: the graphs of training/validation accuracy and training/validation loss tend to converge over time. By comparing these graphs and considering also the accuracies (test accuracy highlighted in bold) and losses shown in Tables 6 and 7 respectively, the similarity of the training, validation and test accuracies can be observed. This clearly highlights the fact that the implemented model is generalizing correctly and does not exhibit overfitting.

**Table 6:** Accuracy with 50 epochs on all datasets

Dataset	Train acc.	Val. acc.	Test acc.	Time(s)
ds1	0.9928	0.9990	0.9990	16038
ds2	0.9931	0.9991	0.9991	8881
ds3	0.9926	0.9982	0.9982	8122
ds4	0.9790	0.9800	0.9980	1537

**Table 7:** Loss with 50 epochs on all datasets

Dataset	Train loss	Val. loss	Test loss	Time(s)
ds1	0.0186	0.0051	0.0051	16038
ds2	0.0190	0.0041	0.0195	8881
ds3	0.0184	0.0048	0.0048	8171
ds4	0.0569	0.0582	0.0099	1537

Further experiments were performed but they did not lead to remarkable improvements. For example, we tried epoch numbers greater than 50 and investigated other regularization techniques such as *L*_1_ and *L*_2_ regularizations without any improvement.

**Fig. 11:**
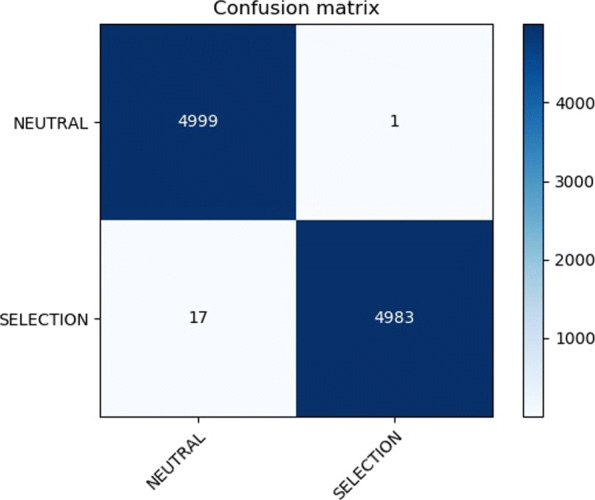
Confusion matrix of the test set on the ds3 dataset using 50 epochs

Let us consider Neutral and Selection examples as positive and negative examples respectively. The *Confusion Matrix* (CM) is used to evaluate the performance of an algorithm. In our case (binary classification), the CM consists of two rows and two columns. Let *A* denote such matrix: in the first row, *A*_11_ (called True Positives *TP*) counts the number of Neutral examples correctly classified while *A*_12_ (called False Negatives *FN*) denotes the number of Neutral examples wrongly classified as Selection. Similarly, in the second row *A*_21_ (False Positives *FP*) and *A*_22_ (True Negatives *TN*) denote the number of Selection examples wrongly and correctly classified respectively. Figure 11 presents the confusion matrix for dataset ds3 using 50 epochs. The results are clearly in line with the values shown in Tables 6 and 7.

Based on the confusion matrix, other metrics such as Precision and Recall can also be computed. The *Precision* is the percentage of observations correctly predicted over the total of observations predicted as positive, $\frac {TP}{TP+FP}$. The *Recall* (also call *sensitivity*) is the percentage of positive observations correctly predicted over the total number of positive observations, $\frac {TP}{TP+FN}$. Table 8 provides the accuracies together with other metrics of all datasets on the test set. The results clearly show that the model is accurate, sensitive and very precise.

**Table 8:** Evaluation metrics using 50 epochs on all datasets

Dataset	Accuracy	Precision	Recall
ds1	0.9990	1.0000	0.9979
ds2	0.9990	0.9996	0.9985
ds3	0.9982	0.9998	0.9966
ds4	0.9990	0.9988	0.9993

Note that the validation/test accuracies are sometimes higher than the training accuracies (especially in ds4). The reason could be related to the fact that the model generalizes very quickly after just a few epochs. In fact the binary images have more 0 than 1 and relevant features necessary to have a clear distinction between neutral and selection are extracted just after a few epochs. Moreover, in Deep Neural Networks, models with initial random weights in some situations could provide similar performance as trained models, see [31]

## Application to real data

**Fig. 12:**
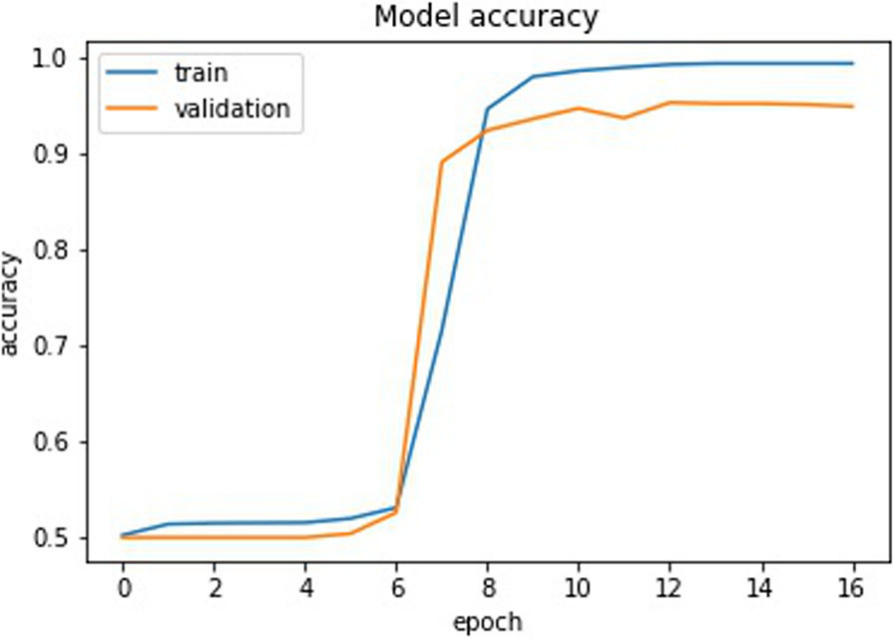
Accuracies with 50 epochs on the ds5 datasets. Training done with early stopping

**Fig. 13:**
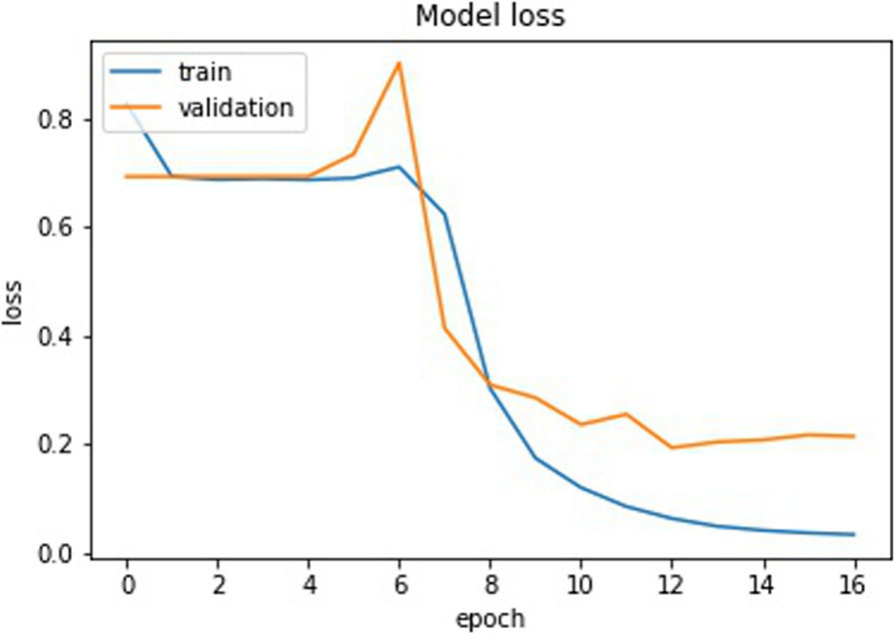
Losses with 50 epochs on the ds5 datasets. Training done with early stopping

**Fig. 14:**
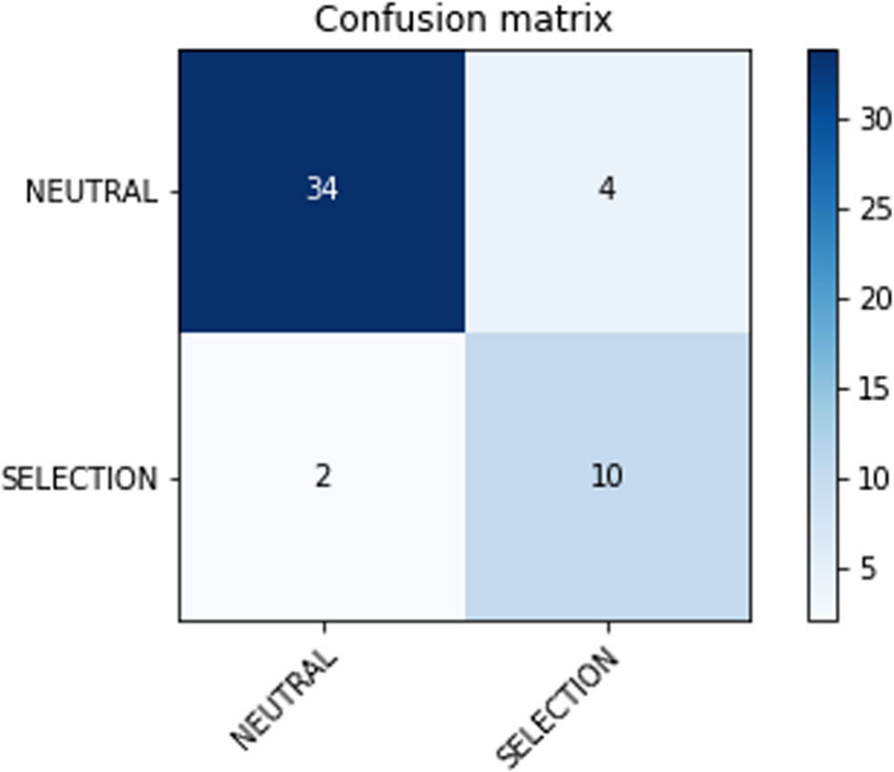
Confusion matrix on the real dataset using 50 epochs and earling stopping

As a proof of principle, we tested our CNN using a real dataset. We chose a clear example of a selective sweep in the SLC24A5 gene (variant rs1426654, Chromosome 15:48134287; Ensembl.org), which was identified as responsible for skin pigmentation loss in human populations of European ancestry, see [32, 33]. Using the variant data from the 1000 Genome Project, we selected the genomic region from position 48000000 to 48500000 on chromosome 15 for 24 individuals sampled in Tuscany (Italy). As the signature left by the selective sweep in this gene spans more than 100 kb, we adjusted our CNN by training it with a new set of simulated windows of 10,000 base pairs generated as in the experiment before but setting an effective population size of 10,000 and a mutation rate of 2E10-8. The training set included 3910 images (1990 as selection and 1920 as neutral), whereas both the training and the validation set included 500 images of each type (dataset ds5). We trained the CNN in Fig. 7 using the Adam optimizer with a learning rate of 0.001. The training was done with early stopping, i.e., the training stops if there is no improvement in the validation accuracy after three epochs. The trend of the accuracy and loss on both training and validation is shown in Figs. 12 and 13 respectively. Note that, contrary to the training described in the Experiments section in which after the first few batches of the first epoch the model is already able to distinguish neutral and selection, the model struggles in the first 7 epochs. Only from the 8th epochs onwards the model starts extracting relevant features necessary to distinguish neutral from selected images. This is probably due to fact that images of 10000x48 contain more information than images of 1000x48. Table 9 recaps the accuracies and losses on the training, validation, and test sets for simulated genomic images. We converted the real genomic data into 50 images of 10000 rows and 48 columns using a custom python script (vcf2raster.py) and categorized the windows between position 48100000 and 48220000 as selection (12 windows) while the remaining 38 windows before and after those positions were labeled as neutral (38 windows). Besides the window including the causative variant rs1426654, the other genomic windows were labeled as under selection if nucleotide diversity was below 0.00003 substitutions per site. This labeling implies that the identification of the regions flanking the causative variant and characterized by a similar genetic variation pattern are also of interest. Our training model categorized the real genomic windows with 88% accuracy, 72% precision, and 84% recall, as shown in the confusion matrix in Fig. 14. Although the performance on the real data already appears satisfactory, additional refinement of the parameters in the simulation step to make it fully compatible with the real data (e.g., including the past demographic history of the studied population) would surely improve it further. Moreover, a combination of CNN trained with different size windows could be used to narrow down the region under selection around the causative variant.

**Table 9:** Accuracies and Losses for Training, Validation and Test on images of sizes 10000x48. Training done with 50 epochs and early stopping

Dataset	Accuracy	Loss
Training	0.9949	0.0340
Validation	0.9500	0.2148
Test	0.9538	0.1744

## Conclusion

In this paper we investigated how to identify patterns of neutral or selection model in genomic sequences. Portions of genomes of a population were represented as images and Convolutional Neural Networks were applied for their classification.

After various experiments with different hyperparameters, we found that it was possible to obtain an acceptable accuracy even with a limited number of epochs. The model was also trained on data generated with realistic parameters and provides good prediction performance on portions of genomes taken from real populations.

As future work we plan to design and train Convolutional Neural Networks that are able to perform prediction under neutral and different selection profiles on portions of genomes taken from simulated data and test the networks on real populations.

## Data Availability

The datasets generated and analysed during the current study are available in the datasets repository, datasets.

## Footnotes

1 Site frequency spectrum (SFS): the distribution of allele frequencies in a population sample.

2 How to generate the dataset is in the directory Dataset_generation available at Experiments.

3 The code is available at Experiments.

## References

[1] Buffalo V, Coop G (2019). The linked selection signature of rapid adaptation in temporal genomic data. Genetics.

[2] Lässig M, Mustonen V, Walczak AM (2017). Predicting evolution. Nat Ecol Evol.

[3] Nielsen R (2005). Molecular signatures of natural selection. Annu Rev Genet.

[4] Blekhman R, Man O, Herrmann L, Boyko AR, Indap A, Kosiol C, Bustamante CD, Teshima KM, Przeworski M (2008). Natural selection on genes that underlie human disease susceptibility. Curr Biol.

[5] Trucchi E, Benazzo A, Lari M, Iob A, Vai S, Nanni L, Bellucci E, Bitocchi E, Raffini F, Xu C (2021). Ancient genomes reveal early andean farmers selected common beans while preserving diversity. Nat Plants.

[6] Horscroft C, Ennis S, Pengelly RJ, Sluckin TJ, Collins A (2019). Sequencing era methods for identifying signatures of selection in the genome. Brief Bioinforma.

[7] Booker TR, Jackson BC, Keightley PD (2017). Detecting positive selection in the genome. BMC Biol.

[8] Stephens ZD, Lee SY, Faghri F, Campbell RH, Zhai C, Efron MJ, Iyer R, Schatz MC, Sinha S, Robinson GE (2015). Big data: astronomical or genomical?. PLoS Biol.

[9] Koumakis L (2020). Deep learning models in genomics; are we there yet?. Comput Struct Biotechnol J.

[10] Eraslan G, Avsec ž, Gagneur J, Theis FJ (2019). Deep learning: new computational modelling techniques for genomics. Nat Rev Genet.

[11] Zou J, Huss M, Abid A, Mohammadi P, Torkamani A, Telenti A (2019). A primer on deep learning in genomics. Nat Genet.

[12] Torada L, Lorenzon L, Beddis A, Isildak U, Pattini L, Mathieson S, Fumagalli M (2019). Imagene: a convolutional neural network to quantify natural selection from genomic data. BMC Bioinformatics.

[13] Michie D, Spiegelhalter DJ, Taylor C (1994). Machine learning. Neural Stat Classif.

[14] LeCun Y, Bengio Y, Hinton G (2015). Deep learning. Nature.

[15] Murphy KP (2012). Machine Learning: a Probabilistic Perspective.

[16] Albawi S, Mohammed TA, Al-Zawi S (2017). Understanding of a convolutional neural network. 2017 International Conference on Engineering and Technology (ICET).

[17] Druzhkov P, Kustikova V (2016). A survey of deep learning methods and software tools for image classification and object detection. Patt Recogn Image Anal.

[18] Fadja AN, Lamma E, Riguzzi F (2018). Vision inspection with neural networks. RiCeRcA@ AI* IA.

[19] Bottou L. Stochastic gradient descent tricks. In: Neural Networks: Tricks of the Trade. Springer: 2012. p. 421–36.

[20] Phansalkar V, Sastry P (1994). Analysis of the back-propagation algorithm with momentum. IEEE Trans Neural Netw.

[21] Khirirat S, Feyzmahdavian HR, Johansson M (2017). Mini-batch gradient descent: Faster convergence under data sparsity. 2017 IEEE 56th Annual Conference on Decision and Control (CDC).

[22] Srivastava N, Hinton G, Krizhevsky A, Sutskever I, Salakhutdinov R (2014). Dropout: a simple way to prevent neural networks from overfitting. J Mach Learn Res.

[23] Goodfellow I, Bengio Y, Courville A, Bengio Y (2016). Deep Learning, vol. 1.

[24] Hudson RR (2002). Generating samples under a wright–fisher neutral model of genetic variation. Bioinformatics.

[25] Ewing G, Hermisson J (2010). MSMS: a coalescent simulation program including recombination, demographic structure and selection at a single locus. Bioinformatics.

[26] Schrider DR, Kern AD (2015). Inferring selective constraint from population genomic data suggests recent regulatory turnover in the human brain. Genome Biol Evol.

[27] Kern AD, Haussler D (2010). A population genetic hidden markov model for detecting genomic regions under selection. Mol Biol Evol.

[28] Schrider DR, Kern AD (2018). Supervised machine learning for population genetics: a new paradigm. Trends Genet.

[29] Kingman JFC (1982). The coalescent. Stoch Process Appl.

[30] Sutskever I, Martens J, Dahl G, Hinton G. On the importance of initialization and momentum in deep learning. In: Int Conf Mach Learn: 2013. p. 1139–47.

[31] Saxe AM, Koh PW, Chen Z, Bhand M, Suresh B, Ng AY (2011). On random weights and unsupervised feature learning. Icml.

[32] Altshuler D, Donnelly P, Consortium IH (2005). A haplotype map of the human genome. Nature.

[33] Szpak M, Xue Y, Ayub Q, Tyler-Smith C (2019). How well do we understand the basis of classic selective sweeps in humans?. FEBS Lett.

